# Optimized Collection Protocol for Plasma MicroRNA Measurement in Patients with Cardiovascular Disease

**DOI:** 10.1155/2016/2901938

**Published:** 2016-09-20

**Authors:** Chi-Sheng Wu, Fen-Chiung Lin, Shu-Jen Chen, Yung-Lung Chen, Wen-Jung Chung, Cheng-I Cheng

**Affiliations:** ^1^Molecular Medicine Research Center, Chang Gung University School of Medicine, Tao-Yuan, Taiwan; ^2^Division of Cardiology, Department of Internal Medicine, Linkou Chang Gung Memorial Hospital, Tao-Yuan, Taiwan; ^3^Division of Cardiology, Department of Internal Medicine, Kaohsiung Chang Gung Memorial Hospital, Kaohsiung, Taiwan

## Abstract

*Background*. Various microRNAs (miRNAs) are used as markers of acute coronary syndrome, in which heparinization is considered mandatory therapy. Nevertheless, a standard method of handling plasma samples has not been proposed, and the effects of heparin treatment on miRNA detection are rarely discussed.* Materials and Method*. This study used quantitative polymerase chain reaction (qPCR) analysis to investigate how storage temperature, standby time, hemolysis, and heparin treatment affect miRNA measurement in plasma samples from 25 patients undergoing cardiac catheterization.* Results*. For most miRNAs, the qPCR results remained consistent during the first 2 hours. The miRNA signals did not significantly differ between samples stored at 4°C before processing and samples stored at room temperature (RT) before processing. miR-451a/miR-23a ratio < 60 indicated < 0.12% hemolysis with 100% sensitivity and 100% specificity. Pretreatment with 0.25 U heparinase I recovered qPCR signals that were reduced by* in vivo* heparinization.* Conclusions*. For miRNA measurement, blood samples stored at RT should be processed into plasma within 2 hours after withdrawal and should be pretreated with 0.25 U heparinase I to overcome heparin-attenuated miRNA signals. The miR-451a/miR-23a ratio is a reliable indicator of significant hemolysis.

## 1. Introduction

Cardiovascular disease (CVD) includes coronary heart disease, cardiomyopathy, hypertensive heart disease, and heart failure. Risk factors for CVD include advanced age, male gender, high blood pressure, smoking, family history of CVD, and family history [1–7]. Although the incidence and mortality rate of CVD are both declining in high-income countries [1], coronary artery disease remains a leading cause of death worldwide [8]. Additionally, CVD may present as acute coronary syndrome (ACS), which has a high mortality rate. Pathologic processes involved in ACS include vascular inflammation, rupture of coronary artery plaques, platelet activation, and subsequent myocardial necrosis. Therefore, many studies have attempted to identify novel biomarkers for identifying patients at high risk of CVD. One proposed biomarker is microRNA (miRNA) [9, 10].

MicroRNA is a noncoding small RNA that is 21–23 nucleotides in length. In plants, animals, and some viruses, small RNA functions as RNA silencer by modifying posttranscriptional regulation [11]. Diseases known to be regulated by miRNA include cancer [12–16], obesity [17, 18], and various other diseases of the nervous system [19], the immune system [20], and the heart [21]. MicroRNA can be sampled from body fluids such as serum, plasma, saliva, and urine. Since miRNA can be collected and detected extracellularly, a major benefit of using miRNA detection for disease diagnosis is its noninvasiveness. Specifically, recent studies indicate that circulating miRNA is a useful biomarker of various diseases [22], including ACS [23, 24].

Cell matrix will be released after erythrocytes rupture due to either physical stress, or red blood cell- (RBC-) specific or RBC-abundant miRNAs are present in hemolyzed blood samples. Hemolysis can potentially affect the accuracy of miRNA quantification in a blood sample. Although studies have shown that expressions of miR-451a and miR-16 in RBCs are detectable in hemolyzed blood samples [25, 26], no studies have thoroughly investigated the potential use of miRNA as an indicator of hemolysis.

In ACS patients, miRNA detection is routinely performed because altered miRNA is associated with ACS risk and outcome [27–32]. However, none of the pioneering studies in the use of miRNA detection have comprehensively discussed sample preparation. For example, reported standby times and storage temperatures of plasma samples during transport from the emergency department to the laboratory vary widely. No information regarding differential patterns of miRNA within the uncertain collection time is available. According to established guidelines, heparin treatment is essential for ACS patients [33], and heparin is known to affect accuracy in detecting miRNA signals [34–37]. Although a previous study showed that heparinase can improve quantitative real-time polymerase chain reaction (qPCR) signals [37], the optimal heparinase dose for qPCR has not been determined.

Therefore, this study developed a multiplex qPCR system for simultaneously screening 18 miRNA targets and determined the optimal miR-451a/miR-23a ratio for predicting hemolysis in plasma samples. A literature review shows that this study is the first report of a standardized procedure for clinical measurement of miRNA in plasma samples from CVD patients and the first to determine the optimal heparinase dose for qPCR.

## 2. Materials and Methods

### 2.1. Clinical Sample Collection

This study was approved by the Institutional Review Board of Kaohsiung Chang Gung Memorial Hospital (102-1790A3). The recruitment criteria were age of 30–70 years, clinical indications for elective cardiac catheterization for ischemic heart disease or heart failure, and written informed consent. Exclusion criteria were hemoglobin less than 12 g/dL, pregnancy, peritoneal dialysis or hemodialysis for end stage renal disease, acute myocardial infarction, contraindications for heparinization, and unstable hemodynamic condition. Clinical data collection included clinical indication for cardiac catheterization and demography.

**Figure 1:**
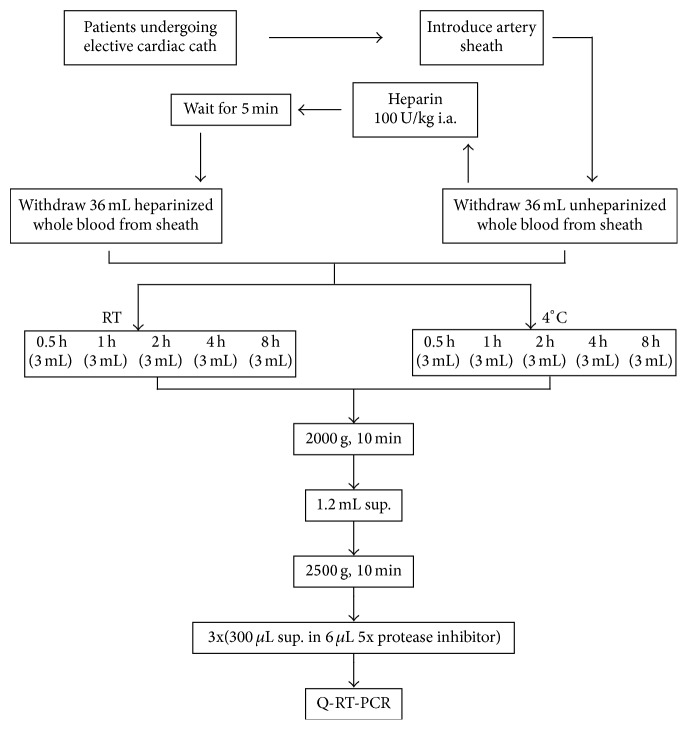
Flowchart of plasma sample collection procedure. Plasma samples were collected at the catheterization laboratory as described in Materials and Methods.

During cardiac catheterization, the radial artery was cannulated with a 6 Fr artery sheath, and 36 mL of unheparinized blood was withdrawn from the sheath. Five minutes after administration of heparin 100 U/Kg through the artery sheath, another 36 mL of heparinized blood was withdrawn. For the coronary angiogram, a cardiac catheter was inserted to ensure an even distribution of heparin in the circulatory system. Next, 6 mL of heparinized or unheparinized blood was dispensed into a 10-mL EDTA K2 tube (BD Vacutainer, Ref. 367525) and kept still at either room temperature (RT) or at 4°C for the time intervals shown in Figure 1. The tube containing the blood sample was centrifuged at 2,000 ×g for 10 minutes. The plasma was then aspirated to another 10 mL centrifuge tube and centrifuged at 2,500 ×g for 15 minutes. Finally, 300 *μ*L of clear plasma was pipetted into a 1.5 mL eppendorf containing 6 *μ*L of protease inhibiter (Roche, Cat. number 11836145001) and stored at −80°C.

### 2.2. Generation of Hemolyzed Plasma Samples

Next, 1 mL unheparinized whole blood derived from some patients was aspirated from the EDTA K2 tube into an eppendorf tube. Varying degrees of hemolysis were induced by vigorous manual shaking until the color of the sample could be matched to the hemolysis color card (see Supplementary Fig.  1A in Supplementary Material available online at http://dx.doi.org/10.1155/2016/2901938). The hemolyzed samples (Supplementary Fig.  1B) were then processed into plasma samples as described above and stored at −80°C.

### 2.3. RNA Preparation and Reverse Transcription

Total RNA from 300 *μ*L of plasma samples was subjected to miRNA extraction with miRNeasy minikit (QIAGEN, GmbH, Hilden, Germany). Briefly, 700 *μ*L of QUIzol reagent was added to the plasma sample, and the sample was allowed to stand at RT for 5 minutes. Next, 1 nM of synthetic cel-miR-39 RNA (5′-CGAUGGGCAGCUAUAUUCACCUUG-3′) was added into the mixture as the spike-in control to monitor the RNA extraction and qPCR processing. Then, 140 *μ*L of chloroform (Merck & Co., Inc.) was added into the sample, mixed well for 15 seconds, and left standing for 3 minutes at RT. The upper layer of 550 *μ*L aqueous solution was aspirated by centrifugation at 15,000 ×g at 4°C for 15 minutes and then thoroughly mixed with 825 *μ*L of ethanol. The samples were further eluted through the microcolumn and washed with RWP and RPE buffer. Finally, total RNA was dissolved in 30 *μ*L of RNase-free water. To convert the detected miRNA into its corresponding cDNA, 5.4 *μ*L of total RNA, 75 nM of 20 miRNA primers mix, 0.5 mM dNTP, 2 U RNaseout, and 120 U Superscript III (Invitrogen, CA) were used for reverse transcription reaction in a total reaction mixture of 12 *μ*L. The processing program was 16°C for 30 minutes; 49 cycles of 20°C for 30 seconds, 42°C for 30 seconds, and 50°C for 1 second; and 72°C for 10 minutes. Reverse transcription products were stored at −20°C.

### 2.4. qPCR Assay

In qPCR assay of 8 *μ*L miRNA, 0.5 *μ*L of a 5-fold dilution of RT product was used as a template. The template was mixed with 4 *μ*L 2x Master Mix (Applied Biosystems, Foster City, CA), 0.25 M universal reverse primer, 0.2 M gene-specific primers, and 0.125 mM TaqMan probe (Applied Biosystems, Foster City, CA). The qPCR conditions (QuantStudio™ 12K Flex Real-time PCR System, Applied Biosystems, Foster City, CA) were 95°C for 10 minutes; 45 cycles of 95°C for 15 seconds and 60°C for 30 seconds; and a dissociation stage.

### 2.5. miRNA Data Analysis

The cycle threshold (Ct) value was calculated by determining the cycle number at which the change in fluorescence intensity crossed the threshold of 0.05. For each sample, the delta Ct was calculated by subtracting the Ct of the sample from the Ct of cel-miR-238. The normalized delta Ct was converted to the miRNA copy number as the copies per uL of plasma.

### 2.6. Heparinase I Usage

To evaluate whether heparinase reverses heparin-related effects on miRNA measurement, heparinase I (H2519, SIGMA-ALDRICH, USA) doses of 0.5 U, 0.25 U, 0.025 U, and 0.0025 U were added into reverse transcription reaction mixes. Briefly, 5.4 *μ*L of the RNA samples derived from heparinized blood was incubated with different doses of heparinase I, 2 U of RNase out, and 1.25 mM MgCl_2_ at 25°C for 1 hour. The RT reaction procedure described above was then performed.

### 2.7. Statistical Analysis

In the storage condition validation group, the variation between 0 h and 2 h was analyzed in each patient by Mann–Whitney test. A *P* value of <0.05 was considered statistically significant. In each patient, the effects of various heparinase dosages administered at RT and at 4°C were compared by ANOVA.

## 3. Results

### 3.1. Patient Characteristics

Table 1 lists the demographic characteristics of the cohort of 25 patients enrolled in this study. The patients had a mean age of 62.0 ± 6.6 years, and 76% (19) of the patients were male. One patient underwent cardiac catheterization to evaluate the etiology of heart failure, and 24 patients underwent cardiac catheterization to evaluate the severity of coronary artery disease.

### 3.2. Specificity of Primer and Probe for Candidate miRNA

Since both nonspecific and background signals can interfere with qPCR, the TaqMan probe specificity with its correlated synthetic cDNA was tested in each of the 18 candidate miRNA targets in this study (Supplementary Fig.  2) to determine the miRNA signal with the best specificity. Cel-miR-238 was used as the spike-in control for monitoring RNA extraction and qPCR detection in plasma samples during the experiment. The primers were designed specifically for the 18 candidate miRNA targets, which were selected because they are known to be associated with CVD. After qPCR assay, the quantification cycle (Ct) value was converted to the copy number (10^6^ copies of each cDNA used as template for qPCR). Supplementary Fig.  2 shows the PCR results, which indicated that each probe had high specificity and high affinity with its own cDNA template. The PCR results indicated that all gene-specific primers and probes were suitable for detecting all candidate miRNAs in our clinical samples.

### 3.3. Hemolysis Test

**Figure 2:**
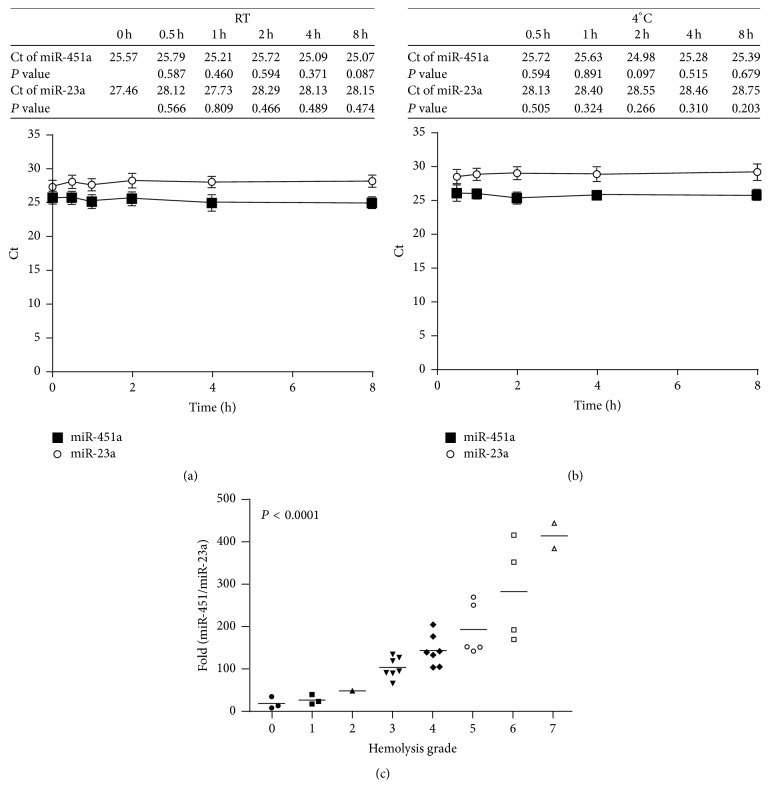
Expressions of miR-425a and miR-23a represent the hemolysis status of clinical samples. The qPCR analysis revealed that expressions of miR-451a and miR-23a stored at (a) RT or (b) at 4°C were stable from 0 h to 8 h. The Ct values shown in the tables indicate the qPCR results for five individuals at different time points. The *P* value indicates the significance of each time point compared with time 0 h. (c) Manual hemolysis test was performed in 32 differential hemolytic plasma samples (see Materials and Methods). Hemolytic grade was defined by hemolysis card (Supplementary Fig.  1A). The miR-425a/miR-23a ratio increased as hemolysis grade increased.

Hemolysis of RBC in clinical samples interferes with the Ct value of qPCR. Additionally, miR-451a expression is associated with hemolysis whereas miR-23a is constant in hemolyzed blood samples [25, 38]. Therefore, this study investigated whether the miR-451a/miR-23a ratio is a good marker of significant hemolysis. First, an artificial mechanical method was used to generate eight different hemolysis grades (grades 0 to 7) from blood samples from four subjects. Supplementary Fig.  1B shows the plasma samples prepared from hemolyzed blood samples. Expressions of miR-451a and miR-23a in these plasma samples were then measured by qPCR. Figures 2(a) and 2(b) show that expressions of miR-451a and miR-23a, respectively, remained stable in unhemolyzed blood samples kept at RT or at 4°C for varying durations.  Figure 2(c) shows that, in further comparisons with other plasma samples of varying hemolysis grades kept at RT for 0 h or 2 h in the same study subjects, a high miR-451a/miR-23a ratio correlated with a high grade of hemolysis. Since hemoglobin 1 g/L [5, 39] is considered mild hemolysis, this study defined significant hemolysis as a hemolysis grade of >0.12%. A receiver operating characteristics curve analysis showed that the miR-451a/miR-23a ratio had an area under the curve value of 1.0 (*P* < 0.001), which indicated that this ratio is a good hemolysis marker. When the cut-off point for the miR-451a/miR-23a ratio was set to 60, both the sensitivity and the specificity were 100% (Supplementary Table  1).

### 3.4. Sample Storage at RT Is Better Than Storage at 4°C

**Figure 3:**
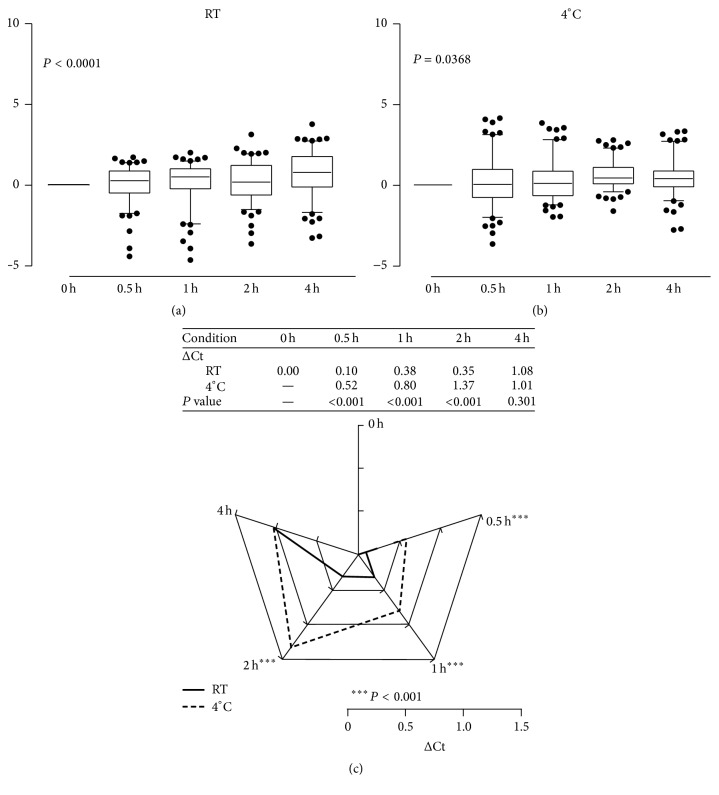
Plasma storage condition test. The miRNA expressin patterns at time 0 h, 0,5 h, 1 h, 2 h, and 4 h for storage at (a) RT and (b) at 4°C. (c) Average change in Ct value in five clinical samples of plasma stored for varying durations ranging from 0.5 h to 4 h at RT and at 4°C. From 0.5 h to 2 h, the delta Ct was more stable at RT than at 4°C. The scale indicates the delta Ct value of the qPCR result in comparison with time 0.

To determine the optimal storage condition for plasma samples used for qPCR detection, plasma samples from five patients (P03, P04, P08, P10, and P11) were used as the training group for qPCR. In each sample, the delta Ct value and average of the 18 miRNA targets were normalized to each Ct value at time 0 at RT (Figure 3(a)) and at 4°C (Figure 3(b)). The results showed that storage time significantly affected the qPCR Ct value under both the RT and 4°C conditions (*P* < 0.0001 and *P* = 0.0368, resp.). These results indicate the need to consider plasma storage conditions when performing qPCR data analysis. To determine the best storage condition, the delta Ct value relative to time 0 of each miRNA was calculated for samples P03, P04, P08, P10, and P11.  Figure 3(c) shows that the RT and 4°C conditions significantly differed (*P* < 0.0001) at 0.5 h, 1 h, and 2 h but not at 4 h. The delta Ct value adjusted to time 0 revealed that the change was smaller at RT than at 4°C before 4 h. These results showed that, for plasma samples processed within 2 h, those stored at RT yield more reliable qPCR results compared to those stored at 4°C.

### 3.5. Expression of miRNA Stored at RT for 2 h

**Figure 4:**
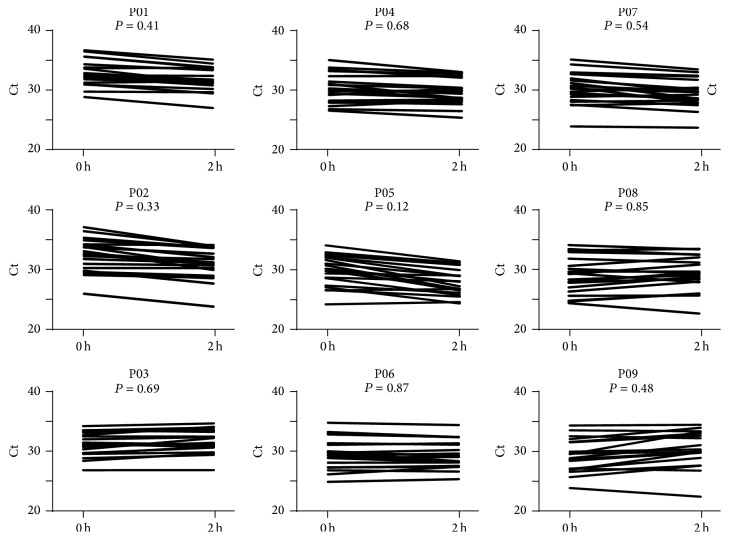
Comparison of samples stored at RT for 0 h and at RT for 2 h. The qPCR results at time 0 and at 2 h were compared in nine individual plasma samples.

This study further evaluated whether samples stored at RT for 2 h were suitable for miRNA detection. Plasma samples derived from nine patients were used as a test cohort to verify the changes in qPCR signals between 0 h and 2 h.  Figure 4 shows that the qPCR signals of 18 miRNA samples between 0 h and 2 h did not significantly differ as a whole in each patient as a whole. However, the statistical data in Table 2 show a significant reduction in the expressions of two miRNA targets (miR-15b-5p and miR-30e-5p) from 0 h to 2 h whereas the largest difference in delta Ct in the three miRNA targets was less than 1.4. These data indicate that although some miRNA targets degraded after storage at RT for 2 h, most targets remained stable.

### 3.6. Heparinase I Improves qPCR Efficiency in Plasma Samples

**Figure 5:**
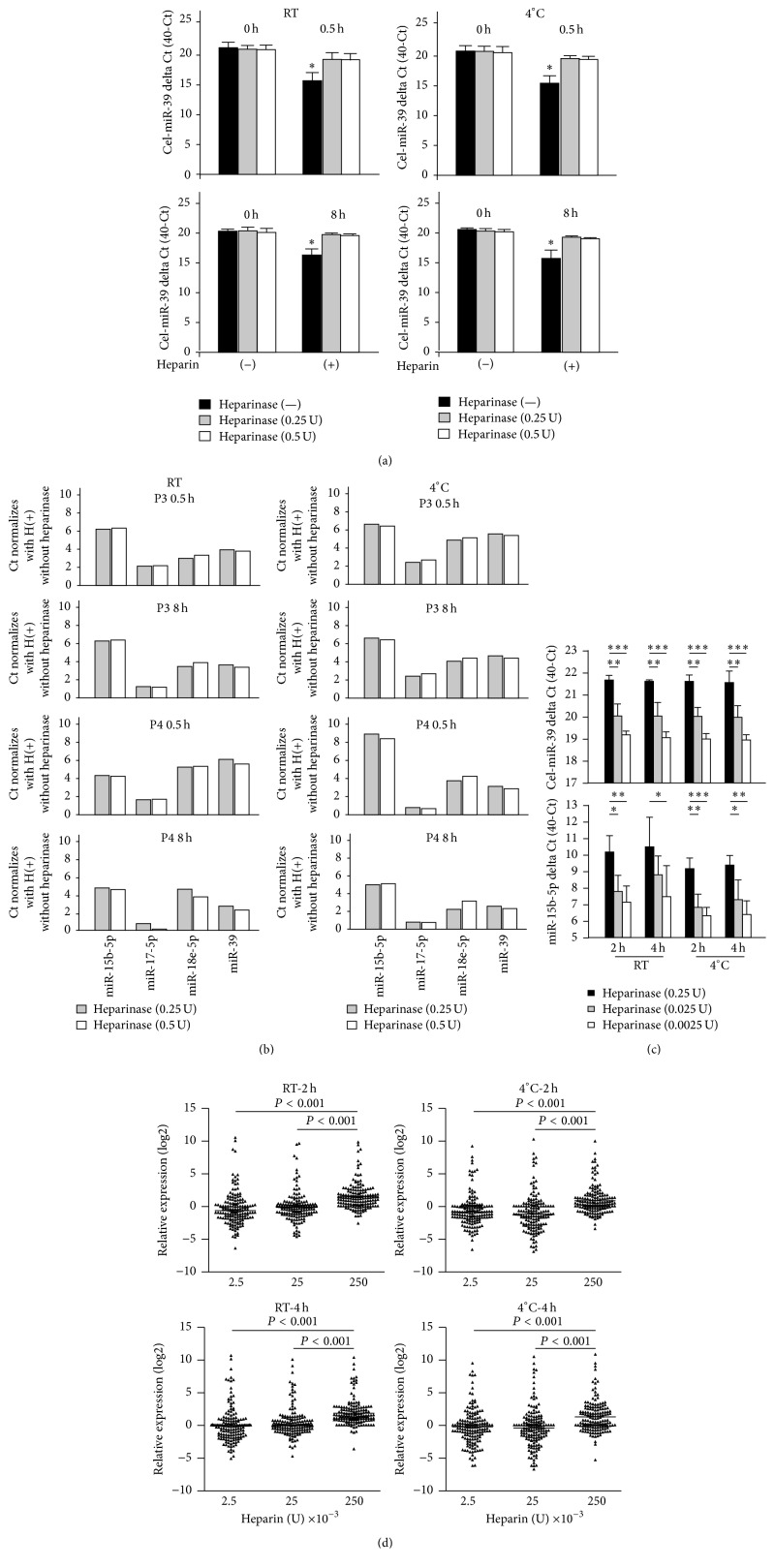
Heparinase I treatment improved Ct values of qPCR signals of clinical samples. (a) The qPCR signal was enhanced by treatment with 0.5 U and 0.25 U of heparinase I in spike-in control cel-miR-39 in four individual plasma samples stored for 0.5 h (top) or at 8 h (bottom) at RT or at 4°C. (b) Two individual plasma samples (P03 ad P04) treated with 0.5 U and 0.25 U heparinase I showed similar qPCR detection results for four independent miRNA targets. (c) Heparinase I treatment improved the qPCR signal. For cel-miR-39 (top), a dose-dependent effect of heparinase I was obsercved in samples stored for 2 h or 4 h at RT or at 4°C. Similar results were observed in four individual samples of human miR-15b-5p. (d) A heparinase I dose of 0.25 U significantly improved the qPCR signal in samples stored for 2 h or 4 h at RT or at 4°C in one-way ANOVA. ^*∗∗∗*^ *P* < 0.001; ^*∗∗*^ *P* < 0.01; ^*∗*^ *P* < 0.05.

Heparin is commonly used to avoid blood clotting in cardiac catheterization. However, since heparin reportedly reduces qPCR signals, this study investigated whether heparinase I treatment can restore miRNA expression in RNA samples before qPCR. First, the control cel-miR-39 was used as a test indicator for comparing qPCR results.  Figure 5(a) shows that, regardless of whether samples were kept at RT or at 4°C and regardless of whether samples were kept for 0.5 h or for 8 h, treatment with 0.25 U and 0.5 U heparinase I significantly increased heparin-reduced delta Ct by 1.5 and 2.8, respectively. Similar results were observed for miR-15b-5p, miR-17-5p, and miR-18e-5p in plasma samples derived from patients P03 and P04 (Figure 5(b)). Next, this study investigated the dose-dependent effects of heparinase I on plasma samples after* in vivo* heparinization.  Figure 5(d) shows that heparinase I improved the qPCR signals of cel-miR-39 and miR-15b-5p at both RT and 4°C. However, 0.25 U heparinase I obtained a significantly larger delta Ct compared to 0.025 U and 0.0025 U heparinase I. In Figure 5(e), a comparison of the Ct values for all miRNA candidates in the multiplex detection panel shows that the total increase in miRNA was ~2 Ct after treatment with 0.25 U at RT and at 4°C. These results indicate that treatment with 0.25 U of heparinase I obtained a Ct value similar to that in untreated samples.

**Figure 6:**
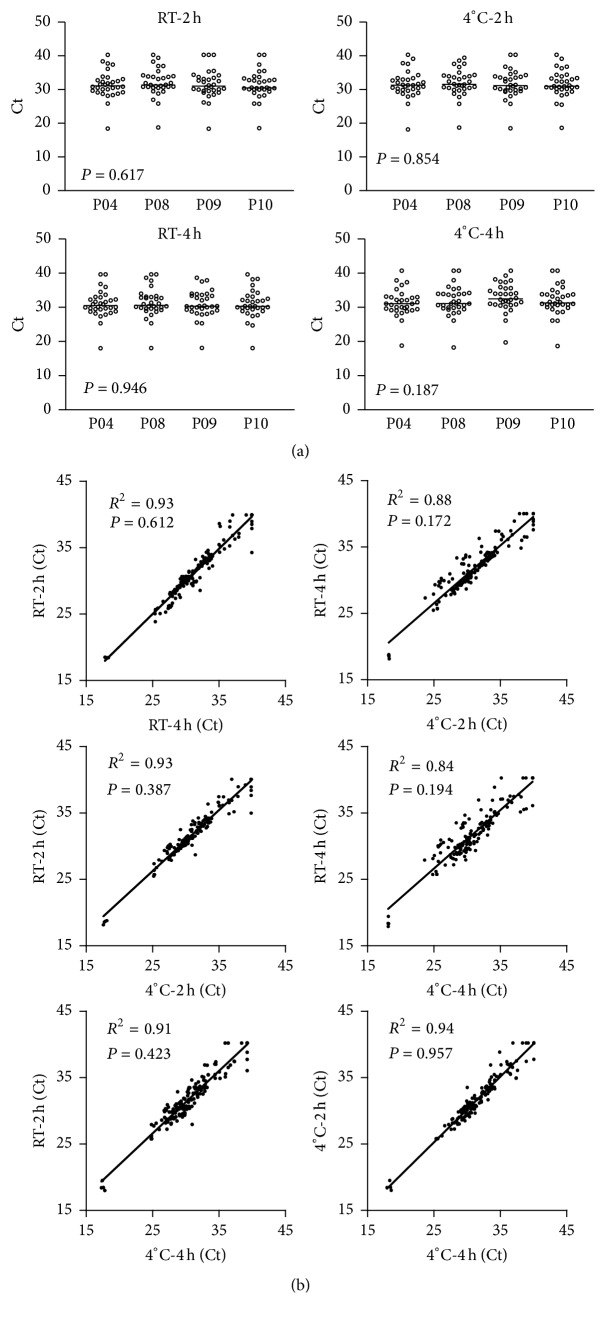
Conservative correlation of miRNA expression induced by heparinase I treatment. (a) In four individuals, treatment with 0.25 U heparinase I induced similar miRNA expression levels in samples stored for 2 h at RT or at 4°C (top). Similar results were observed in samples stored for 4 h (bottom). (b) One-way ANOVA showed that miRNA expression induced by 0.25 U heparinase I treatment significantly correlated with storage time and storage temperature.

Expressions of 18 miRNA in individuals treated with 0.25 U heparinase were similar.  Figure 6(a) shows that, in four individuals, expression of miRNA did not differ at RT or at 4°C after 2 h or 4 h. To simulate the common clinical scenario of delayed sample processing, correlation coefficients between 2 h and 4 h were calculated for 0.25 U heparinase I at both RT and 4°C.  Figure 6(b) shows that the correlation coefficients were 0.84–0.93 between different standby times and temperatures (*P* < 0.05). The experimental results show that that treatment with 0.25 U heparinase I improves the Ct value of plasma samples stored for 2 h or for 4 h at RT or at 4°C.

## 4. Discussion

Because both plasma samples and serum samples are easily obtained, circulating miRNA is now considered emerging biomarkers for many diseases [25]. Many factors, for example, anticoagulant treatment, can affect the signal obtained in miRNA measurement [36]. Therefore, the plasma processing procedure is an important issue because procedural differences can obtain very different test results. Procedural differences may also explain the wide diversity of biomarkers used for the same disease in previous publications. Blood samples extracted must be processed into plasma or serum before miRNA measurement. However, miRNA in whole blood samples or processed plasma stored at RT or even refrigerated may start to degrade [40]. Another problem is that blood samples may not be adequately protected in emergency wards and in some clinical scenarios. Hence, the objective of this study was to develop and validate a standard procedure for processing heparinase I. Experiments showed that miRNA stored at RT remains stable for 2 h after the sample is taken and that 0.25 U heparinase I can recover the qPCR signal of miRNA when the signal is attenuated by heparin treatment.

### 4.1. Optimized Clinical Procedure for Sample Collection and Processing

In clinical practice, plasma sampling and RNA extraction are performed using diverse methods [25, 26]. Additionally, the time needed for withdrawing and processing blood samples may vary widely in different clinical settings. These confounding factors may then affect miRNA measurements. Our experiments compared qPCR signals at different time points and under different temperature conditions. The experiments showed that most target miRNAs stored at RT remained stable for 2 h, and their qPCR results were comparable to those stored at 4°C for 2 h. These data indicate that blood samples extracted from patients in an emergency department can be stably stored at RT for 2 hours. However, miR-15b-5p and miR-30e-5p measurements significantly differed between 0 h and 2 h. Notably, since only nine clinical samples were analyzed, significant differences may have resulted from individual variability. Therefore, these data should be interpreted cautiously until the degradation kinetics of specific miRNAs are clarified in further experiments.

### 4.2. Effect of Heparinase on Recovery of Heparin-I Induced Attenuation of qPCR Signals

Heparin is a widely used medication for treating CVD (including ACS) and for preventing blood clotting in some clinical procedures such as percutaneous coronary intervention and cardiac surgery. Plasma derived from CVD patients contains coagulation cascade, which is a clotting factor. Anticoagulants can cause inaccurate miRNA measurements through mechanisms that are poorly understood. Therefore, the* in vivo* experiments in this study investigated the effects of heparin treatment on miRNA expression. The Ct values of miRNA targets derived from plasma samples treated with or without* Flavobacterium* heparinase I were compared. The signals significantly improved in the control, cel-miR-39, and target miRNAs, and the Ct was observed almost 5 cycles earlier in samples treated with 0.25 U or with 0.5 U heparinase I and stored at RT or at 4°C after treatment. The qPCR results were superior to those reported in a previous study [41]. The effects were also consistent after different standby times and after storage at different temperatures before sample processing. Notably, the qPCR signal can be improved with a* Flavobacterium* heparinase I dose as low as 0.25 U. This dose is much smaller than the required* Bacteroides* heparinase dose reported in a previous work [42] but obtains a similar magnitude of recovery. The experimental results show that* Flavobacterium* heparinase I treatment improves qPCR signals attenuated by heparinization.

### 4.3. Clinical Implications

This study showed that, for miRNA quantification, whole blood samples should be processed into plasma within 2 hours after withdrawal and should be stored at RT rather than at 4°C. Additionally,* in vitro* experiments should be performed to investigate the degrading kinetics of a specific miRNA before clinical application. Finally, plasma samples should be treated with 0.25 U of heparinase I to recover* in vivo* heparin-related delay of Ct in the qPCR, and the ratio of miR-451a/miR-23a should be assessed at regular intervals to evaluate hemolysis.

## 5. Conclusions

Before processing into plasma for miRNA measurement, whole blood samples should be stored at RT for no longer than 2 h after withdrawal. Pretreating samples with 0.25 U heparinase I can recover miRNA signals attenuated by heparin. A reliable indicator of severe hemolysis is miR-451a/miR-23a > 60.

## Supplementary Material

(A) The plasma sample color of various hemolysis grades as indicated.
(B) The color of hemolyzed samples derived from 4 study subjects after manual hemolysis procedure.

## Figures and Tables

**Table 1:** Patient demographics.

Clinical characteristics	Patient number (%)

Age (years)	62.0 ± 6.6
Male	19 (76%)
Hypertension	20 (80%)
Diabetes	10 (40%)
Atrial fibrillation	4 (16%)
Heart failure	6 (24%)
Stroke	5 (20%)
Coronary artery disease	5 (20%)
Chronic kidney disease	5 (20%)
Indications for cardiac catheterization	
Evaluation of coronary artery disease	24 (96%)
Evaluation of heart failure	1 (4%)

**Table 2:** Expression of miRNA (ct value) at 0 h and 2 h.

miRNA	0 h	2 h	*P* ^*∗*^
	Median ± SD	Median ± SD	
hsa-miR-15b-5p	30.55 ± 0.98	29.75 ± 1.07	0.0315^†^
hsa-miR-17-5p	29 ± 1.08	29.43 ± 1.19	0.3401
hsa-miR-19a-3p	29.85 ± 0.96	31.39 ± 1.25	0.3865
hsa-miR-20a-5p	32.98 ± 1.05	32.09 ± 1.08	0.1135
hsa-miR-21-5p	32.29 ± 0.87	32.43 ± 0.97	0.7962
hsa-miR-24-3p	28.37 ± 1.17	28.06 ± 1.26	0.8633
hsa-miR-27a-3p	30.04 ± 0.96	29.7 ± 1	0.2581
hsa-miR-27b-3p	30.35 ± 0.99	30.59 ± 1.01	1
hsa-miR-30c-5p	33.07 ± 0.98	32.78 ± 0.88	0.7962
hsa-miR-30e-5p	34.25 ± 1.11	32.84 ± 1.06	0.0315^†^
hsa-miR-145-5p	32.01 ± 0.8	31.31 ± 1.12	0.2581
hsa-miR-150-5p	29.84 ± 0.86	30.31 ± 0.61	0.2973
hsa-miR-199a-3p	31.51 ± 1.51	30.78 ± 1.95	0.7304
hsa-miR-210	33.6 ± 1.19	33.5 ± 1.15	0.3401
hsa-miR-221-3p	29.06 ± 1.25	29.82 ± 1.43	0.6665
hsa-miR-222-3p	31.12 ± 0.89	30.65 ± 0.92	0.1615
hsa-miR-320a	28.26 ± 0.86	28.85 ± 0.8	0.2224
hsa-miR-423-5p	28.81 ± 1.11	28.52 ± 1.07	0.4363

^*∗*^Mann–Whitney *U* test.

^†^Statistically significant.

## Abbreviations

qPCR: Quantitative real-time polymerase chain reaction
ACS: Acute coronary syndrome
CVD: Cardiovascular disease.

## Additional Points

*Research Highlights*. (1) Different storage conditions for plasma samples before qPCR analysis of miRNA were compared. (2) The miR-451a/miR-23a ratio was evaluated for use as a hemolysis indicator in plasma samples. (3) The heparinase dose that obtained the best qPCR results in clinical samples was determined.
