# Supplementary material for: Optimized Collection Protocol for Plasma MicroRNA Measurement in Patients with Cardiovascular Disease
Source: Biomed Res Int. 2016 Sep 20;2016:2901938. doi: 10.1155/2016/2901938 (PMC5048034; doi:10.1155/2016/2901938)
Supplement: Supplementary file 1 — (A) The plasma sample color of various hemolysis grades as indicated. (B) The color of hemolyzed samples derived from 4 study subjects after manual hemolysis procedure. [file 2901938.f1.docx]

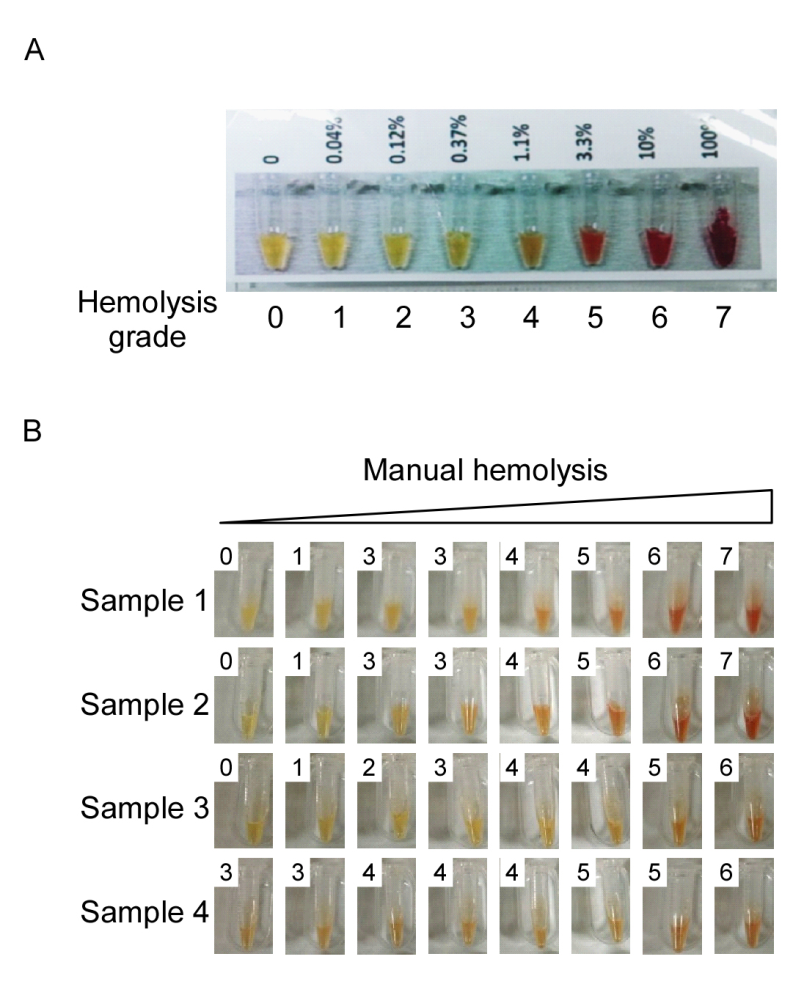


Supplementary Figure 1. Definition of Hemolysis grade. A. Hemolysis card used to define the hemolysis degree in this study. B. Plasma samples of manual hemolysis test. The number indicate the hemolytic grade of the plasma sample determined by hemolysis card ,A.


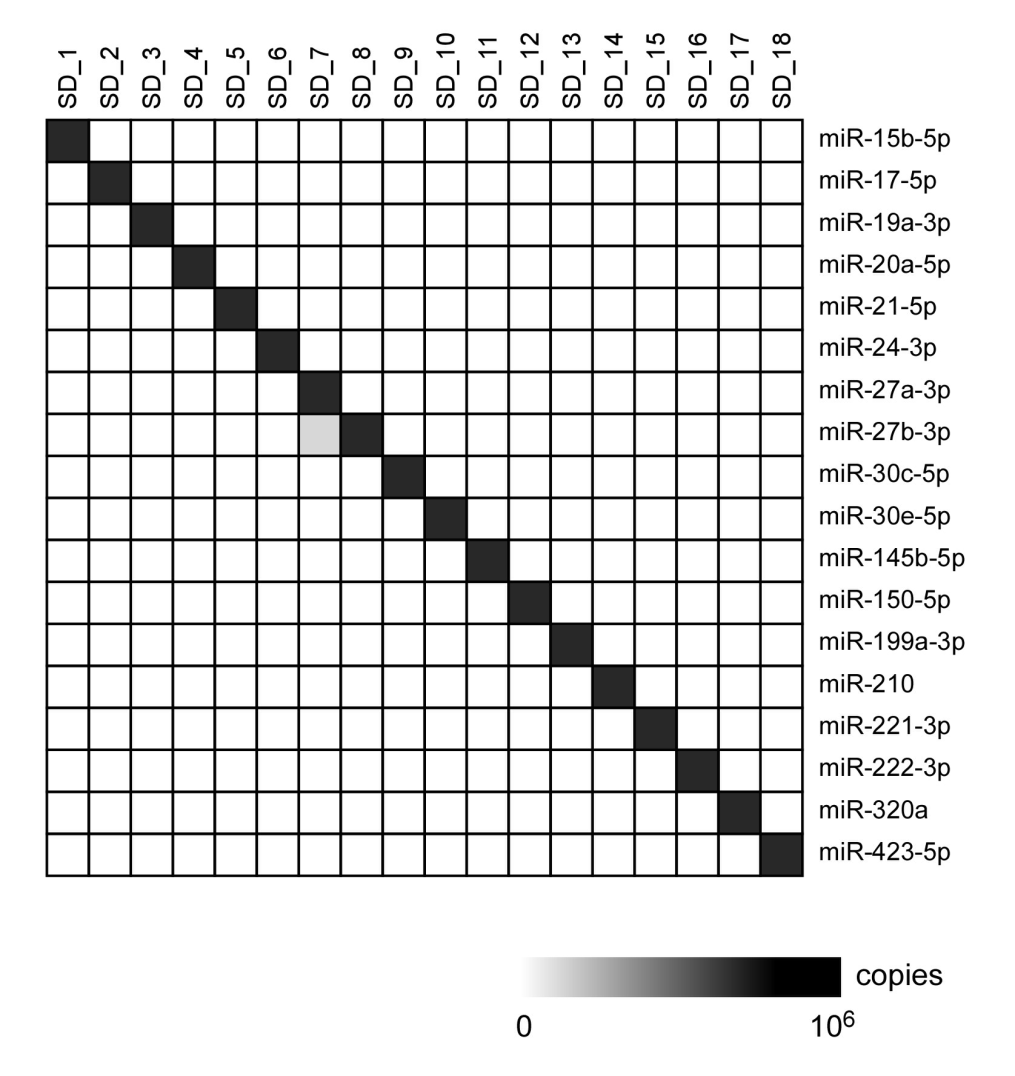


**Supplementary Figure 2. Specificity assay of TaqMan probes used in this study.** SD_1 to SD_18 indicate synthetic cDNA of target microRNA genes from miR-15b-5p to miR-423-5p list on of the right side chessboard. Black square indicate TaqMan probe yield the best amount of copies. MiR-27b-3p show little crossed reactive with miR-27a-3p cDNA and yield 1.8X10^5^ copies.

**Supplementary Table 1. Diagnostic of manual hemolysis test.**

|  | | |
| --- | --- | --- |
| Positive if Greater Than or Equal To^a^ | Sensitivity | 1 - Specificity |
| 10.6965 | 1.000 | 1.000 |
| 14.1937 | 1.000 | .857 |
| 18.8651 | 1.000 | .714 |
| 23.9925 | 1.000 | .571 |
| 32.4608 | 1.000 | .429 |
| 40.4689 | 1.000 | .286 |
| 47.1154 | 1.000 | .143 |
| 60.3375 | 1.000 | .000 |
| 81.1982 | .960 | .000 |
| 93.7697 | .920 | .000 |
| 96.3867 | .880 | .000 |
| 102.3726 | .840 | .000 |
| 107.1527 | .800 | .000 |
| 114.8186 | .760 | .000 |
| 125.8650 | .720 | .000 |
| 132.4938 | .680 | .000 |
| 136.1014 | .640 | .000 |
| 139.3127 | .600 | .000 |
| 142.7705 | .560 | .000 |
| 144.2575 | .520 | .000 |
| 148.9987 | .480 | .000 |
| 154.0237 | .440 | .000 |
| 163.0249 | .400 | .000 |
| 175.0716 | .360 | .000 |
| 186.3313 | .320 | .000 |
| 200.1130 | .280 | .000 |
| 228.9081 | .240 | .000 |
| 260.9119 | .200 | .000 |
| 311.0582 | .160 | .000 |
| 367.6864 | .120 | .000 |
| 399.0999 | .080 | .000 |
| 428.6824 | .040 | .000 |
| 443.6431 | 0 | .000 |
